# Full-field strain distribution in hierarchical electrospun nanofibrous poly-L(lactic) acid/collagen scaffolds for tendon and ligament regeneration: A multiscale study

**DOI:** 10.1016/j.heliyon.2024.e26796

**Published:** 2024-02-24

**Authors:** Alberto Sensini, Olga Stamati, Gregorio Marchiori, Nicola Sancisi, Carlo Gotti, Gianluca Giavaresi, Luca Cristofolini, Maria Letizia Focarete, Andrea Zucchelli, Gianluca Tozzi

**Affiliations:** a Department of Complex Tissue Regeneration and cell Biology-Inspired Tissue Regeneration, MERLN Institute for Technology-Inspired Regenerative Medicine, Maastricht University, Maastricht, the Netherlands; b Department of Industrial Engineering, Alma Mater Studiorum—Università di Bologna, Bologna, Italy; c ESRF The European Synchrotron, Grenoble, France; d Surgical Sciences and Technologies, IRCCS Istituto Ortopedico Rizzoli, Bologna, Italy; e Advanced Mechanics and Materials – Interdepartmental Center for Industrial Research (CIRI-MAM), Alma Mater Studiorum—University of Bologna, Bologna, Italy; f Health Sciences and Technologies—Interdepartmental Center for Industrial Research (HST-ICIR), Alma Mater Studiorum—Università di Bologna, I-40064, Ozzano dell'Emilia, Bologna, Italy; g Department of Chemistry 'G. Ciamician' and National Consortium of Materials Science and Technology (INSTM, Bologna RU), Alma Mater Studiorum—Università di Bologna, Bologna, Italy; h Centre for Advanced Manufacturing and Materials, School of Engineering, University of Greenwich, Chatham Maritime, United Kingdom

**Keywords:** Hierarchical scaffolds, Electrospinning, MicroCT, In situ mechanical tests, Digital volume correlation, Tendons and ligaments

## Abstract

Regeneration of injured tendons and ligaments (T/L) is a worldwide need. In this study electrospun hierarchical scaffolds made of a poly-L (lactic) acid/collagen blend were developed reproducing all the multiscale levels of aggregation of these tissues. Scanning electron microscopy, microCT and tensile mechanical tests were carried out, including a multiscale digital volume correlation analysis to measure the full-field strain distribution of electrospun structures. The principal strains (ε_p1_ and ε_p3_) described the pattern of strains caused by the nanofibers rearrangement, while the deviatoric strains (ε_D_) revealed the related internal sliding of nanofibers and bundles. The results of this study confirmed the biomimicry of such electrospun hierarchical scaffolds, paving the way to further tissue engineering and clinical applications.

## Highlights

• Electrospun PLLA/Coll-75/25 bundles and hierarchical scaffolds were successfully produced.
• Electrospun hierarchical scaffolds mimic the morphology and mechanical properties ranging natural fascicles to whole tendons and ligaments.
• A microCT in situ protocol was successfully developed to test the mechanical properties of electrospun scaffolds.
• Digital volume correlation allowed measurement of the full-field strain distribution inside electrospun scaffolds.

## Graphical abstract

**Figure:**
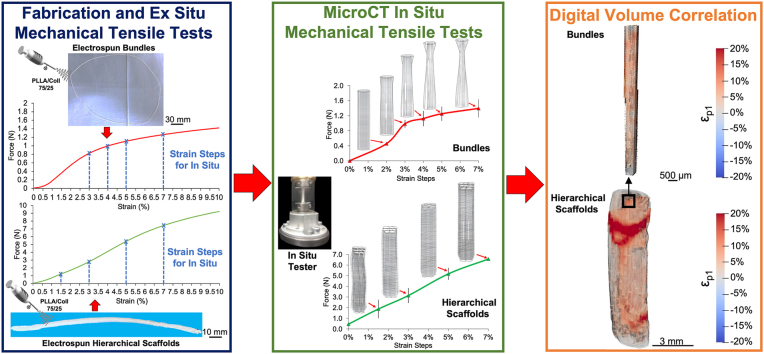

## 1 Introduction

Injuries and ruptures of tendon/ligament (T/L) tissue represent one of the main challenges in modern orthopedics with approximately 30 million new injuries to these tissues worldwide and in constant increment each year [1]. The main cause of these lesions resides the high strains they are subjected to, that often damage their multiscale structure composed of nanometric fibrils of collagen type I, axially aligned and progressively aggregated in different hierarchical levels from the nano-up to the macroscale [2]. This complex morphology leads to non-linear mechanical properties, resulting from the interaction of these hierarchical levels [3]. To address the challenge of T/L regeneration, in the last twenty years tissue engineering has developed complex scaffolds to speed up their regeneration [[4], [5], [6]]. Among the various biofabrication techniques explored, electrospinning is for sure one of the most promising [7,8]. Sophisticated electrospun hierarchical structures, made of resorbable or biostable polymers, were developed to mimic T/L from the collagen fascicles [[9], [10], [11], [12], [13], [14], [15]], up to the whole tissue level [[16], [17], [18], [19], [20]], showing promising outcomes in enhancing cell proliferation and extracellular matrix (ECM) production by maintaining high morphological and mechanical biomimicry. Specifically, mechanical strains are a key aspect in the design of biomimetic scaffolds and it has been widely demonstrated how those contribute to guiding cells for the production of new ECM [[21], [22], [23]]. For this reason, several studies attempted to identify the two-dimensional strain patterns developed on the surface of natural or synthetic tissues mainly via digital image correlation (DIC) [24]. DIC investigations on T/L tissues were mostly focused on the human Iliotibial and Achilles tendons or on the Anterior Cruciate Ligament [[25], [26], [27], [28], [29], [30]]. From the biofabrication side instead, DIC was used on electrospun mats for tissue engineering and enthesis (tendon to bone attachment) regeneration [[31], [32], [33], [34]], allowing to define their strain gradients. However, DIC is constrained to the measurement of superficial strains on the tested specimen and, in complex scaffolds architectures, this is not sufficient to describe how local mechanics relates to their internal microstructure under load. In fact, electrospun scaffolds, and mostly their more complex hierarchically organized versions [[16], [17], [18], [19], [20]], are composed of millions of nanofibers sliding with each other when a load is applied (as T/L collagen fibrils do) and the pattern of such intimate strain-driven interaction is unexplored to date. Moreover, these internal material rearrangements substantially influence the morphological changes of cells proliferating inside them (both in static but mostly in dynamic conditions) [11,[16], [17], [18]]. To overcome this limitation, Digital Volume Correlation (DVC) was developed [35]. In brief, DVC relies on grayscale recognizable features, typically from x-ray micro computed tomography (microCT) images of materials subjected to progressive loading in situ, to measure volumetric full-field displacement and strain fields. The technique has been widely employed in musculoskeletal research [36]. Focusing on the T/L tissue instead, due to the high resolution and contrast required, microCT studies were performed mostly in static conditions by dehydrating samples, using contrast agents, or eventually performing phase-contrast synchrotron x-ray images [[37], [38], [39], [40], [41], [42]]. To the authors’ knowledge, only one study has been carried out so far using DVC to study strain distributions in the rat enthesis [43]. Conversely, DVC analyses on electrospun materials and scaffolds are completely unexplored so far, due to the concomitant need of high resolution and the low x-ray absorption of polymeric fibrous materials. Thus, defining a DVC protocol to investigate the full-field strain distribution inside electrospun scaffolds is needed to finely tune their structure and mechanical properties, to optimally guide cells in their morphological/phenotype changes and in the production of new ECM during the early regeneration stages post-implantation.

Considering this background, the study aims at developing and applying the first microCT in situ protocol investigating the multiscale full-field strain distribution of electrospun structures via DVC. Results from single bundles and hierarchical scaffolds are also obtained and presented.

## 2 Materials and methods

### 2.1 Materials

Acid soluble collagen type I (Coll), obtained from bovine skin (Kensey Nash Corporation DSM Biomedical, Exton, USA) and poly-L (lactic) acid (PLLA) (Lacea H.100-E, Mw = 8.4 × 10^4^ g mol^−1^, PDI = 1.7, Mitsui Fine Chemicals, Dusseldorf, Germany) were used. As a solvent system, a mixture of 2,2,2-trifluoroethanol (TFE), 1,1,1,3,3,3-Hexafluoro-2-propanol (HFIP) (Sigma-Aldrich, Staint Louis, USA) were used in a 50:50 (v/v) percentage. To crosslink Coll in the nanofibers, N-(3-Dimethylaminopropyl)-N′-ethylcarbodiimide hydrochloride (EDC), N-hydroxysuccinimide (NHS) and ethanol (Sigma-Aldrich, Staint Louis, USA) were used. The following polymeric blend solution was used: PLLA/Coll-75/25 (w/w) prepared from a 18% (w/v) solution of PLLA and Coll dissolved in TFE:HFIP = 50:50 (v/v).

### 2.2 Scaffolds Fabrication

To mimic the morphology of T/L fibrils and fascicles [2,44,45], PLLA/Coll-75/25 electrospun bundles of aligned nanofibers were produced as previously described [[13], [14], [15]]. To obtain ring-shaped bundles (RB) with a diameter in the range of human fascicle (500–650 μm), an industrial electrospinning machine (Spinbow srl, Bologna, Italy), equipped with a high-speed rotating drum collector (length = 405 mm, diameter = 150 mm; peripheral speed = 19.6 m s^−1^; drum rotations = 2500 rpm) and using an applied voltage of 22 kV, was used. To make easier the detachment of the nanofibers’ mats, the drum was covered with a sheet of polyethylene coated paper (Turconi S.p.A, Italy). The polymeric solution was spun with four metallic needles (internal diameter = 0.51 mm, Hamilton, Romania), via polytetrafluoroethylene tubes (Bola, Germany), using a feed rate of 0.5 mL h^−1^ imposed by a syringe pump (KD Scientific 200 series, IL, United States).

**Fig. 1:**
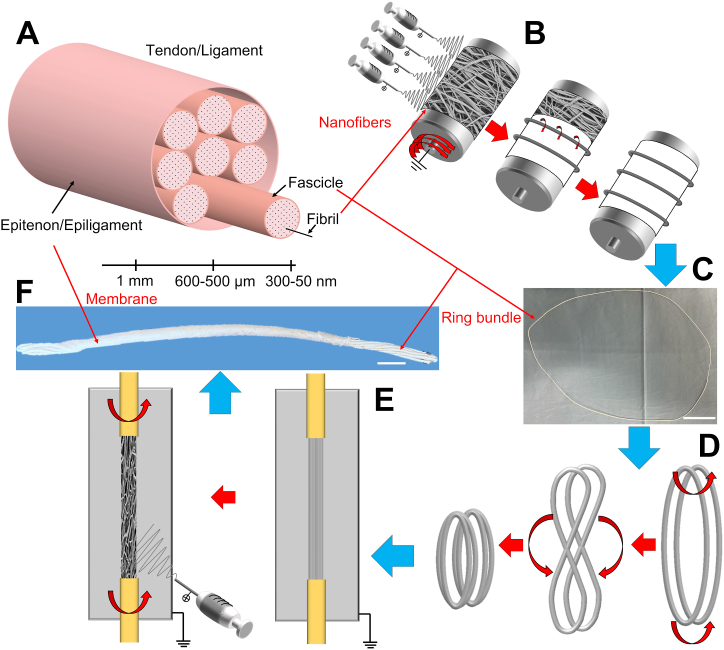
Electrospun scaffolds production. A) Hierarchical structure of tendons and ligaments. B) Electrospun ring bundles production. C) Example of a ring bundle (scale bar = 30 mm). D) Assembly of an EHS. E) Electrospun membrane production. F) Example of a final EHS obtained (scale bar = 5 mm).

The needles-collector distance was 200 mm while the sliding spinneret, supporting the needles, had an excursion of 180 mm, with a sliding speed of 1500 mm min^−1^. The electrospinning session was set at 2 h at room temperature and with a relative humidity of 20–30%. After the electrospinning session, the mat was cut in circumferential stripes of 45 mm, wrapped up and pulled off the drum obtaining RB of aligned nanofibers (Fig. 1B and C). To mimic the hierarchical structure of T/L [2,44,45] (Fig. 1A), hierarchical electrospun scaffold (EHS) (Fig. 1F) were assembled. Each RB was twisted in the middle and bent over itself (Fig. 1D). Then each assembly, composed of two folded RB, was covered with an electrospun epitenon/epiligament-like membrane, as previously described [17,18,46]. In brief, a second electrospinning machine (Spinbow srl, Bologna, Italy) made by an high-voltage power supply (FuG Elektronik GmbH, Schechen, Germany) and a syringe pump (KD Scientific Legato 100, Illinois, USA) was employed to electrospin the solution. The two folded RB were placed in a custom-made setup, equipped with a flat plate aluminum collector, able to rotate the bundles during the electrospinning session. To produce the membrane, RB were maintained in a static position but alternated with rotation sessions (5 sessions of approximately 10 rpm for 30 s every 20 min of stasis) (Fig. 1E). The PLLA/Coll-75/25 solution and the electrospinning parameters were the same as previously described [14,18]. The scaffolds were finally crosslinked with a crosslinking solution of EDC and NHS 0.02 M in 95% ethanol, following a consolidated procedure [14].

### 2.3 SEM investigation

To visualize, at high-resolution, bundles and EHS surfaces, from the nano-up to the microscale, a Scanning Electron Microscopy (SEM) investigation was carried out. Before the analysis, samples were gold-sputtered and then imaged with a SEM at 10 kV (SEM, Phenom Pro-X, PhenomWorld, Eindhoven, Netherlands). To measure the diameters of 200 nanofibers (both for RB and EHS membranes; magnification = 8,000x) ImageJ [47] was used allowing also to compute the nanofiber diameter distribution. RB and EHS diameters were measured via an optical microscope (Axioskop, Zeiss, Pleasanton, CA, United States) connected to a camera (AxioCam MRc, Zeiss, Pleasanton, CA, United States) as mean and SD of 20 measures. The nanofiber orientation was investigated with the Directionality plugin of ImageJ [48]. This plugin of ImageJ allows to quantify the number of nanofibers within a given range of angles from the axis and, to be consistent with our previous validated method, a Local Gradient Orientation modality was employed [49]. For both bundles and EHS membrane, the analysis was performed on five images (magnification = 8,000x) along the scaffold's axis and the results reported as mean and standard deviation between the five images.

### 2.4 Mechanical characterization

To investigate the mechanical properties of the electrospun scaffolds and to set up the strain steps for the later in situ test, a mechanical tensile characterization of samples was performed with a material testing machine (Mod. 4465, Instron, Norwood, United States) equipped with a ±100 N load cell (Instron, Norwood, United States). The testing machine worked under displacement control to obtain a strain rate of 0.33% s^−^^1^. This strain-rate was chosen to ensure consistency with the in situ tests described in paragraph 2.5. For each sample type (i.e., RB and EHS) dedicated capstan grips were used to reduce the stress concentration (see Fig. 3A and B). To guarantee scaffold hydration with their collagen molecules in a physiological environment [14], each specimen was immersed for 2 min in phosphate buffer saline (PBS), before testing. The mechanical performances of RB (n = 5) and EHS (n = 5) were tested using a monotonic ramp to failure (with a procedure adapted from the ASTM D1414 Standard) consistently with our previous study [50]. RB had a gauge length of 176 ± 1 mm while EHS had a gauge length of 90.0 ± 1 mm, caused by the shrinkage of RB after removal from the drum collector and the subsequent crosslinking. The force-displacement curves were converted to stress-strain graphs by calculating two different kinds of stresses. In the first method, the apparent stress, calculated by dividing the force for the cross-sectional area of the specimen measured before the test, was plotted against strain. In the second method instead, the net stress was calculated. This approach allows to determine the mechanical properties of the specimen independently from its internal porosity. The net stress was calculated by dividing the apparent stress for the volume fraction (*v*) of the specimen under investigation. Specifically, *v* was calculated by using the following equation (Eq. 1):

(Eq. 1) $$\upsilon =\frac{w}{\left(L∙A∙\rho \right)}$$

Where w is the weight of the specimen, L is length of the specimen, A is the cross-sectional area of the specimen, ρ is the density of the blend that, considering the density of PLLA (ρ_PLLA_ = 1.25 g cm^−3^) and of the Coll (ρ_Coll_ = 1.34 g cm^−3^), resulted in (Eq. (2)):

(Eq.2) $$\rho =\left(0.75\right)\cdot \rho_{\text{PLLA}}+\left(0.25\right)∙\rho_{\text{Coll}}=1.27g{cm}^{-3}$$

The weight of each specimen was calculated using a precision balance (AS 60/220.R2, Radwag, Pol). The following mechanical properties were then calculated: yield force (F_Y_), yield stress (σ_Y_), yield strain (ε_Y_), elastic modulus (E), failure force (F_F_), failure stress (σ_F_), failure strain (ε_F_), unit work to yield (W_Y_), unit work to failure (W_F_). Moreover, by dividing by half the force of the RB, it was also possible to calculate F_Y_ and F_F_ of one of their branches, to set the strain values for the following in situ test on the single bundles (SB).

The inflection point value of scaffolds was evaluated using a previously consolidated method [15]. This value is defined in literature as the point in the linear region of the stress-strain curve of the specimen under investigation, where the material pass from strain-stiffening to strain-softening. A MATLAB script was developed, based on the csaps function, with the aim of fit a cubic smoothing spline with the mechanical data. A smoothing parameter in the csaps routine was set to 0.7, to guarantee a smooth first and second derivative curves. The inflection point was finallycomputed by locating the zero point of the second derivative [51]. Mean and SD values of inflection point strain (IPε), apparent (IPσ_App._) and net (IPσ_Net_) stress for SB|RB and EHS were calculated.

To investigate the axial and transversal strains of the EHS membranes, 4K movies of the specimens, during the tensile test previously described, were acquired with a camera (12-megapixel, Sony, JAP) synchronized with the testing machine. To allow an accurate measurement of the lengths of interest during the test, rulers were placed on each capstan grip (see Fig. 3E). Before the start of each tensile test, two zero-strain images were acquired as a reference. These images were used to calculate the axial (mean ± SD of 10 measures) and transversal (mean ± SD of 10 measures) length of the external membrane of the EHS under investigation. Then, after the mechanical elaboration of the curves, 5 high-resolution movie frames were selected corresponding to specific levels of strain (i.e. 1.5%, 3%, 5%, 7%, ε_Y_ and ε_F_) of the EHS previously calculated. For each image the axial and transversal length of the membrane were calculated with the same procedure reported for the zero-strain axial and transversal reference images. Finally, the axial (*ε*_*MA*_) and transversal (*ε*_*MT*_) strains of each membrane were calculated as follows (Eq. (Eq.3), (Eq.4))):

(Eq.3) $$\varepsilon_{MA\%}=\frac{l_{MA}-l_{MA0}}{l_{MA0}}∙100$$

(Eq.4) $$\varepsilon_{MT\%}=\frac{d_{MT}-d_{MT0}}{d_{MT0}}∙100$$

Where (*l*_*MA*_*)* is the mean axial length of the membrane corresponding to the investigated percentage of axial strain of the EHS specimen; (*l*_*MA0*_*)* is the mean axial length of the membrane at the zero-axial strain of the EHS specimen; (*d*_*MT*_*)* is the mean transversal diameter of the membrane at the investigated percentage of the axial strain of the EHS specimen; *(d*_*MT0*_*)* is the mean transversal diameter of the membrane at the zero-axial strain of the EHS specimen. All the measurements were taken with ImageJ.

### 2.5 MicroCT in situ protocol

Samples of SB (n = 3) and EHS (n = 3) underwent microCT in situ mechanical tests. The gauge length of the samples was measured by the in situ loading device, while the diameter by using ImageJ on optical microscope images. Each sample was immersed in PBS for 2 min (SB and EHS gauge length = 10 mm) before it was clamped in the microCT loading device (maximum actuator displacement = 5.5 mm; displacement rate = 0.001 mm s^−1^) (MTS in situ tester for Skyscan 1172, Bruker, Belgium). The first two consecutive microCT scans were acquired at the minimum strain allowed by the load cell sensitivity (0.45 N, corresponding to about 2% strain for SB and 0% strain for EHS) and were used to compute the DVC measurement uncertainties [52]. Then, a series of progressive axial strain steps were imposed for SB (i.e. 3%, 4%, 5%, 7%) and for EHS (i.e. 1.5%, 3%, 5%, 7%). Strain levels were reached by imposing axial displacements based on the initial clamp-clamp distance, as measured on radiographic images on coronal and sagittal planes, while the tensile force was measured. At each strain step a 15 min stress-relaxation period was applied followed by a microCT acquisition [53].

SB samples were imaged with an applied voltage of 40 kV and current of 75 μA. The scan orbit was 180° with a rotation step of 0.8° and 4 frames averaged for each rotation angle, resulting in a voxel size of 13 μm and scan duration of ∼17 min. The scanning protocol for EHS samples was the same, except for increasing the voxel size to 9 μm to better discriminate between bundles and membrane. The image reconstruction was carried out with a modified Feldkamp algorithm by using the SkyScanTM NRecon software accelerated by GPU.

The region of interest (ROI) selection and morphometric analysis were all performed using SkyScan CT-Analyser (Skyscan 1172, Bruker, Belgium) software. The ROI extended 6 mm in length, which is 10 mm of gauge length minus 4 mm, to be away from steel clamps, avoiding metal artefacts. Membranes and SB could not be defined in a fully automatic way, by morphological operations, because of the tightening in traction and overlapping grey intensity distributions. For each sample and strain level, the following parameters were measured using the microCT post-processing software (CT-Analyser, Skyscan 1172, Bruker, Belgium).

1. Porosity *Po* (%): percentage of void space inside samples, was calculated by a 3D integrated Analysis (i.e. 3D morphometric parameters integrated for the whole volume) after defining a ROI that wraps the sample and binarizing the image with an automatic thresholding. For the EHS samples, a total (*Tot.Po*) and an internal (*Int.Po*) (i.e. excluding the membrane) porosity were defined (see Fig. S1A);
2. MicroCT-computed cross-sectional area (i.e. *CT.Cr.Ar* in mm^2^): obtained by averaging the areas of the 450 transversal sections of the wrapping ROI described at point 1) net of porosity *Po* to consider resisting material only;
3. SB orientation and tortuosity: calculated from morphological parameters of individual objects that follow the bundles' major development in 3D space (see Fig. S1B). Orientation *θ*(°) is defined as the object (i.e. SB) angle respect to the loading (i.e. vertical axis Z in Fig. S1). It is 0° when parallel, 90° when orthogonal. Tortuosity (*τ*) instead, is defined as the object (i.e. bundle) equivalent length divided by the ROI vertical length (minimum value = 1, when the bundle is perfectly vertically aligned and without crimps) [54]. The same procedure was applied on the eight bundles inside each EHS.

Finally, thanks to the microCT-based morphometric parameters, the in situ strain-stress curves were calculated, considering the cross-sectional area of samples avoiding micro porosities visible from the microCT. In this way the microCT stress *CT*.σ (MPa) was defined as (Eq. (5)):

(Eq.5) $$CT.\sigma =\frac{CT.F}{CT.Cr.Ar}$$

where *CT.F* (N) is the force recorded by the in situ tester during the experiment.

### 2.6 Digital volume correlation

Digital volume correlation was carried out by using the open-source software *spam* [55]. The correlation procedure in *spam* target the measurement of a linear and homogeneous function, Φ, expressed in homogeneous coordinates and consisting of a 4 × 4 deformation matrix. This matrix accounts for 3D affine transformations: translation, rotation, normal and shear strain. The he correlation algorithm is based on a gradient-based iterative procedure, which minimizes the difference between the reference image and the deformed one, the latter being gradually corrected by using a trial deformation function. The convergence criterion is funded on the norm of the deformation function increment between two successive iteration steps, which was set here as: ∥δΦ∥ < 10^−4^. A maximum number of 500 iterations was set as a limit to stop the iterative procedure in case of the convergence criterion was not met.

A total DVC analysis was performed mapping the first scan (i.e., undeformed sample) with each of the remaining scans, as opposed to an incremental analysis which maps two consecutive load steps. To overcome the problem of the progressive large amounts of deformation for a total DVC analysis, for each pair of images an initial non-rigid registration was performed, which measured the overall average displacement, strain and rotation. This initial overall guess was then passed to a local approach, whereby independent cubic sub-volumes (i.e., correlation windows) were defined in the reference image (i.e., undeformed sample) and sought in the deformed image by applying the iterative procedure mentioned above. A Φ was computed in the center of each window, yielding a field of deformation functions that mapped the reference to the deformed image. The size of the correlation windows and the number of measurement points depend on the texture of the imaged samples and define the spatial resolution of the measured field. For SB, a single run with a window size of 36 (i.e. 468 μm) pixels was enough to achieve a well-converged deformation field. For EHS samples, to achieve a good convergence in the local calculations, as well as a high spatially resolved deformation field a two-step approach of local DVC computations was performed with decreasing correlation window sizes from 100 (i.e. 900 μm) to 40 (i.e. 360 μm) pixels. In all cases an overlap of 50% was set between neighboring correlation sub-volumes.

Strains were obtained by extracting only the displacement part of the total fields of Φ and computing the transformation gradient tensor (Eq. (6)):

(Eq. 6) F=I+δu∙δx

on Q8 shape functions linking 2 × 2 × 2 neighboring measurement points. Note that the displacement field was firstly smoothed by applying a 3D median filter of a 1 voxel radius. A polar decomposition of the transformation gradient tensor (Eq. (7)):

(Eq. 7) F=R∙U

yielded the right stretch tensor U and the rotation tensor R for each Q8 element. The finite large-strain framework was used to calculate:

i) the principal strains ε_p1_ and ε_p3_ based on the diagonalization of the right Cauchy-Green deformation tensor (Eq. (8)):

(Eq. 8) $$C=F^{T}∙F=U^{2}$$

ii) the deviatoric strain ε_D_ based on a multiplicative decomposition of the stretch tensor U into a pseudo-isotropic and deviatoric part (Eq. (9)):

(Eq. 9) $$\varepsilon_{D}=\left\|U_{\text{dev}}-I\right\|\;\text{with}\;U_{\text{dev}}={\det\left(F\right)}^{-\frac{1}{3}}∙U$$

The level of uncertainty in the DVC procedure was estimated through a correlation analysis of the zero-strain scans [52]. As already mentioned, at the beginning of each test two scans of the undeformed sample were acquired. DVC was then run with the exact same parameters as the ones for the pair of images during the loading. The mean computed strain uncertainties were for SB: ε_p1_ = 0.2%, ε_p3_ = 0.2% and ε_D_ = 0.3%; for EHS instead: ε_p1_ = 0.3%, ε_p3_ = 0.3% and ε_D_ = 0.6%. DVC maps were overlaid onto microCT stacks using ImageJ and ParaView [56].

### 2.7 Statistical analysis

The significance of differences between the apparent mechanical properties (i.e. forces) for the SB (n = 5), RB (n = 5) and EHS (n = 5) was assessed with an ANOVA 1 unpaired parametric *t*-test with a Tukey post hoc (p > 0.05, ns; p ≤ 0.05, *; p ≤ 0.01; **; p ≤ 0.001, ***; p ≤ 0.0001, ****). The significance of differences of apparent and net mechanical properties, between SB|RB (equal for single and ring bundles) and EHS was assessed with an unpaired parametric *t*-test with Welch's correction. Instead, the comparison between the apparent and net mechanical properties of the same sample (i.e. SB|RB and HNES) was assessed with a ratio paired parametric *t*-test.

## 3 Results and discussion

### 3.1 Morphology of bundles and EHS via SEM

**Fig. 2:**
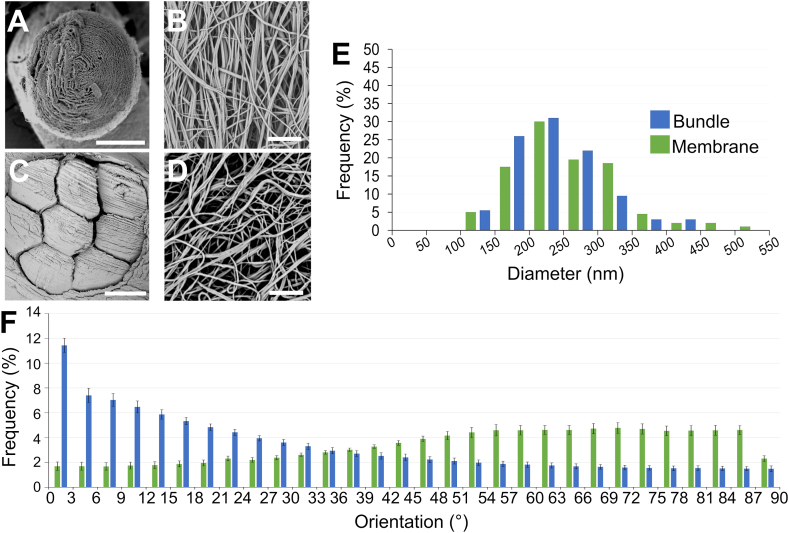
SEM images and morphological investigations of scaffolds. A) Bundle (scale bar = 300 μm; magnification = 500x). B) Nanofibers of the bundle (scale bar = 5 μm; magnification = 8000x). C) EHS (scale bar = 300 μm; magnification = 245x). D) Nanofibers of the membrane (scale bar = 5 μm; magnification = 8000x). E) Nanofibers diameter distribution for the bundles and membranes. F) Orientation of the nanofibers of bundles and EHS membranes. The Directionality histograms show the distribution of the nanofibers in the different directions. An angle of 0° means that the nanofibers were aligned with the axis of the scaffold, while an angle of 90° means that the nanofibers were perpendicular to the scaffold.

In this study, electrospun scaffolds made from a resorbable blend of PLLA/Coll were investigated. These materials were chosen for their broad applications in T/L tissue regeneration [4] and to enhance the scaffolds' bioactivity in biological environments, as collagen type I is the predominant component of T/L ECM [2,3,44,45]. Starting with a top-down approach, EHS (mean cross-sectional diameter = 2.7 ± 0.3 mm; mean length = 90 ± 1.0 mm; mean weight = 96 ± 10 mg) and bundles (mean cross-sectional diameter = 560 ± 93 μm; mean length = 176 ± 1.0 mm; mean weight = 35 ± 5 mg) had a similar morphology (Fig. 2A and C) and thickness of natural T/L reported in literature [2,3,44,45]. The SEM investigation revealed that PLLA/Coll-75/25 nanofibers of bundles (mean cross-sectional diameter = 0.238 ± 0.06 μm) (Fig. 2B) and membranes (mean cross-sectional diameter = 0.258 ± 0.08 μm) (Fig. 2D) were continuous, smooth and without defects such as beads. They also were in the same order of magnitude of T/L collagen fibrils [2,44,45]. The lower dispersion and diameters of the nanofibers of bundles compared with the ones of membranes, were consistent with the higher stretching caused by the drum collector (Fig. 2E). Interestingly, the Directionality analysis revealed a preferential axial orientation of the nanofibers of bundles and a slightly circumferential orientation of the membranes, consistently with our previous work (Fig. 2F) [18]. The circumferential orientation of nanofibers in membranes confirmed the ability of the process to pack and tighten the structure. A relevant number of nanofibers of bundles were oriented in the range of 0°–12° (32.3% ± 2.2% of the total) from the bundle axis with a Gaussian-like distribution. A small number of nanofibers were oriented in the range of 81°–90° (6.0% ± 0.8% of the total). Conversely, the EHS membrane had lower number of nanofibers in the range of 0°–12° (6.8% ± 1.2% of the total) compared with the ones in the range of 81°–90° (16.1% ± 1.24% of the total). This analysis confirmed the morphological biomimicry of the bundles with the T/L fascicles and of the membranes of EHS with the epitenon/epiligament [2,44,45].

**Fig. 3:**
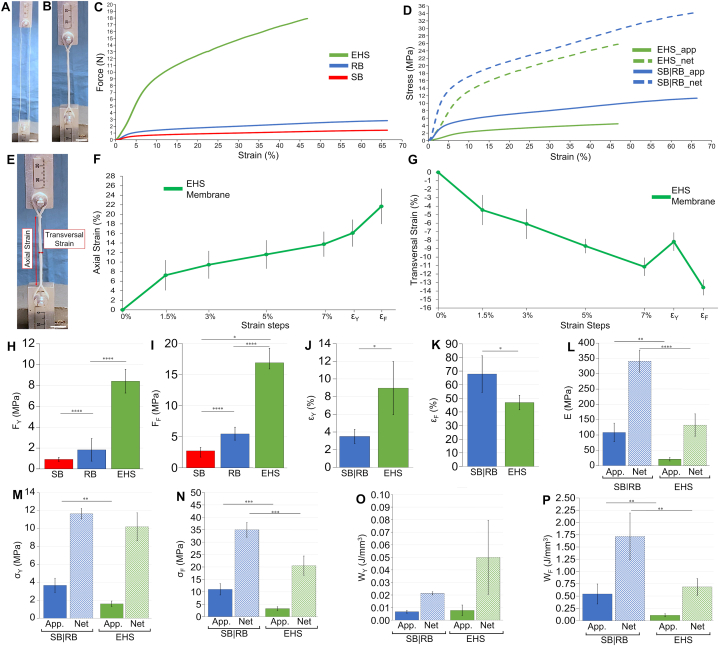
Mechanical tensile tests on bundles and EHS. A) Setup for testing RB (scale bar = 10 mm); B) setup for testing EHS (scale bar = 10 mm); C) typical force-strain curves for SB, RB and EHS; D) typical apparent and net stress-strain curves for SB|RB (same behavior being SB a branch of RB) and EHS; E) example of axial and transversal strains (scale bar = 10 mm); F) mean and SD of axial strain of EHS membranes at the different levels of strain of the in situ test including the yield and failure strain of EHS tensile tests; G) mean and SD of transversal strain of EHS membranes at the different levels of strain of the in situ test including the yield and failure strain of EHS tensile tests; H) yield force of SB, RB and EHS (significance of differences of the ANOVA 1 showed with asterisks); I) failure force of SB, RB and EHS (significance of differences of the ANOVA 1 showed with asterisks); J) yield strain of SB|RB and EHS; K) failure strain of SB|RB and EHS; L) apparent and net elastic modulus for SB|RB and EHS; M) apparent and net yield stress for SB|RB and EHS; N) apparent and net failure stress for SB|RB and EHS; O) apparent and net work to yield for SB|RB and EHS; P) apparent and net work to failure for SB|RB and EHS. (significance of differences reported with asterisks: H, I ANOVA 1 unpaired parametric *t*-test with a Tukey post hoc; J-P unpaired parametric *t*-test with Welch's correction).

### 3.2 Mechanical properties of bundles and EHS

The mechanical properties of SB, RB and EHS, used to set the strain values for the in situ experiments, are reported in Fig. 3 and Tables S2–S4.

Both bundles and EHS showed a nonlinear toe region up to 2% strain, caused by the progressive stretching of the nanofibers under the applied load, followed by a linear elastic region, similar to the nonlinear behavior of fascicles and whole T/L [3,45]. By increasing the hierarchical complexity of scaffolds (i.e. by passing from SB, RB and EHS) their levels of yield force (F_Y_) and failure force (F_F_) progressively increased (Fig. 3H and I). Then, at different levels of strain (SB|RB: ε_Y_ = 3.5 ± 0.11%; EHS: ε_Y_ = 8.97 ± 3.01%), scaffolds showed a ductile region (Fig. 3C and J), partially caused by the bulk material properties and by the breakage of relevant number of nanofibers, up to their failure occurred at ε_F_ = 67.8 ± 13.4% for SB/RB and at ε_F_ = 46.9 ± 5.3% for EHS (Fig. 3C and K). The lower levels of failure strain for EHS were due to the folding procedure to obtain them. These failure strains were higher than those of natural fascicles and T/L [3,43], but can provide a safety factor in case of partial damage of scaffolds, with a relevant work absorption (Fig. 3P), preventing a premature implant failure. The Other mechanical properties (Fig. 3 and Table S1), such as stress (Fig. 3D, M and 3 N), elastic modulus (Fig. 3L) and work to yield and failure (Fig. 3O and P), increased by approximately three times (for SB|RB) and six times (for EHS) passing from the apparent (SB|RB: σ_YApp._ = 3.67 ± 0.80 MPa, σ_FApp._ = 11.0 ± 2.3 MPa, E_App._ = 108 ± 30 MPa, W_YApp._ = 0.007 ± 0.001 J mm^−3^, W_FApp._ = 0.54 ± 0.20 J mm^−3^; EHS: σ_YApp._ = 1.61 ± 0.31 MPa, σ_FApp._ = 3.24 ± 0.77 MPa, E_App._ = 20.7 ± 5.90 MPa, W_YApp._ = 0.008 ± 0.004 J mm^−3^, W_FApp._ = 0.11 ± 0.03 J mm^−3^) to the net ones (SB|RB: σ_YNet._ = 11.7 ± 0.58 MPa, σ_FNet._ = 35.0 ± 3.0 MPa, E_Net._ = 341 ± 30 MPa, W_YNet._ = 0.021 ± 0.001 J mm^−3^, W_FNet._ = 1.72 ± 0.50 J mm^−3^; EHS: σ_YNet._ = 10.2 ± 1.55 MPa, σ_FNet._ = 20.6 ± 3.87 MPa, E_Net._ = 133 ± 37 MPa, W_YNet._ = 0.05 ± 0.03 J mm^−3^, W_FNet._ = 0.69 ± 0.17 J mm^−3^) with statistically significant differences (Fig. 3 and Table S4). In fact, the net mechanical properties consider only the contribution of the volume fraction of the solid material that constitutes the scaffolds (i.e. ν_SB|RB_ = 0.32 ± 0.05; ν_EHS_ = 0.16 ± 0.02), without their internal porosities. Focusing on the net properties, all the mechanical values fell into the range of human fascicles [57] and whole T/L [3,45,58]. As for the inflection point, SB|RB showed values of IPε = 2.12 ± 0.57 %, of IPσ_App._ = 2.47 ± 0.93 MPa and of IPσ_Net_ = 7.74 ± 2.07 MPa, while EHS of IPε = 6.66 ± 3.12 %, of IPσ_App._ = 1.25 ± 0.47 MPa and of IPσ_Net_ = 7.83 ± 2.39 MPa. Moreover, except for a scalable increment in terms of forces with respect to SB|RB, the greater hierarchical complexity of EHS led to a decrement of stress, strain, elastic modulus and works with respect to bundles. This was caused by the increment of the internal adjustments of bundles with load and the higher porosity compared to SB|RB (i.e. for elastic modulus and stress and works). It is worth mentioning that, despite the low strain-rate (0.33 % s^−1^) adopted, the mechanical properties of both SB|RB and EHS fell into the range for natural fascicles and whole T/L with similar dimensions [3,45,58]. Also, their mechanical properties are in line with similar electrospun T/L scaffolds reported in literature [4,[7], [8], [9]]. The strain rate used in this study (0.33 % s^−1^) was set to meet the loading profile achievable with the in situ tensile tester. However, being these scaffolds highly viscoelastic, the application of higher strain rates simulating relevant physiological tasks (such as 10–100 % s^−1^ [59,60] would have produced even more biomimetic mechanical properties [14,17]. The epitenon/epiligament-inspired membranes of EHS successfully enclosed the internal bundles up to their failure (Fig. 3F and G and Table S5) showing a progressive increment of their axial strain at the different levels of EHS strain of the tensile test, in correspondence to the in situ strain steps. The transversal strains instead, showed a progressive reduction, caused by the striction and adjustments of the internal bundles up to 7% of EHS strain, then increasing before EHS ε_Y_ and finally reducing again to EHS ε_F_. The increment in transversal strain in the range 7% - ε_Y_ was due to the tendency of the internal bundles to follow the capstan grips external diameter.

### 3.3 Morphology and mechanics of bundles and EHS via microCT in situ tests

**Fig. 4:**
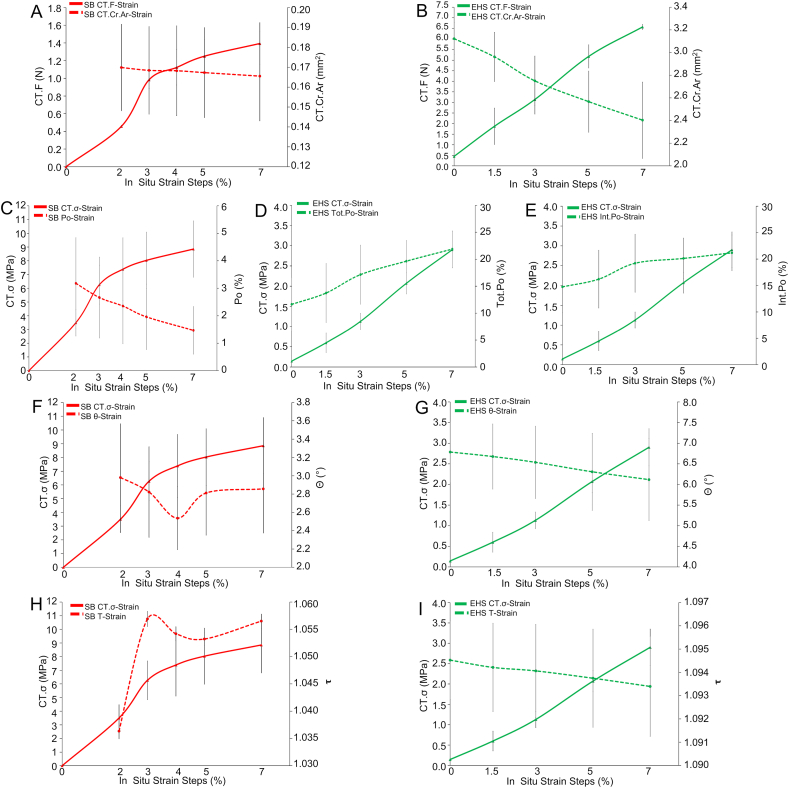
Evolution (mean and SD at the different in situ strain steps) of SB (red lines) and EHS (green lines) morphometric parameters and mechanical characteristics in comparison with the corresponding *CT.F* and *CT.σ*: A) SB *CT.F* and *CT.Cr.Ar*; B) EHS *CT.F* and *CT.Cr.Ar*; C) SB *CT.σ* and *Po*; D) EHS *CT.σ* and *Tot.Po*; E) EHS *CT.σ* and *Int.Po*; F) SB *CT.σ* and *θ*; G) EHS *CT.σ* and *θ*; H) SB *CT.σ* and *τ*; I) EHS *CT.σ* and *τ*.

**Fig. 5:**
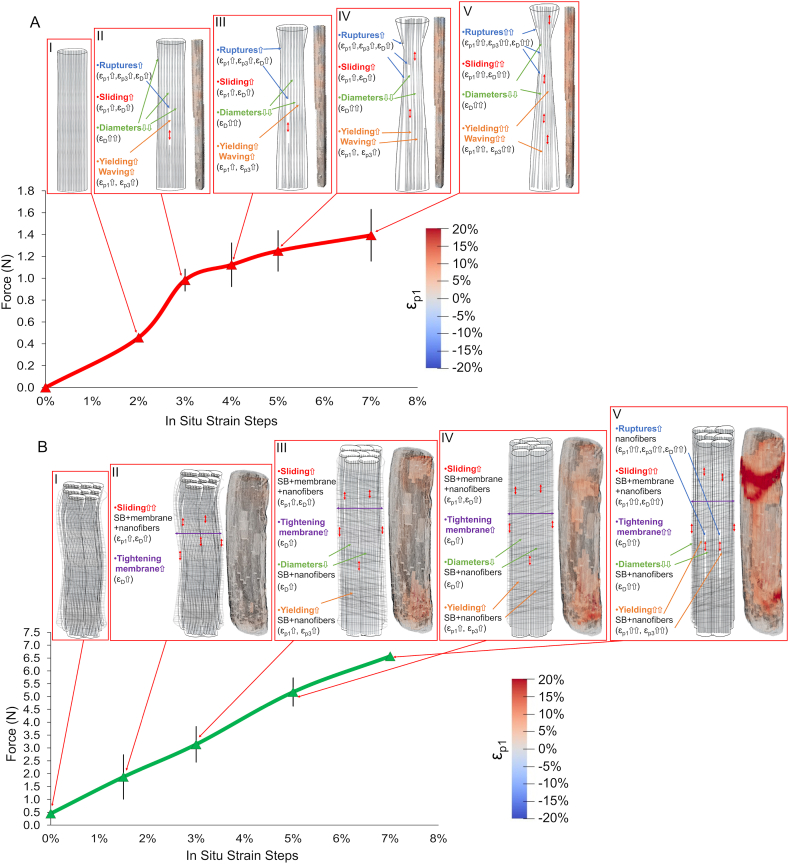
DVC strain evolution as a consequence of nanofibers and scaffolds rearrangement during the different in situ mechanical test. A) Strain evolution of SB at the different strain steps: AI) 2%; AII) 3%; AIII) 4%; AIV) 5%; AV) 7%. B) Strain evolution of EHS at the different strain steps: BI) 0%; BII) 1.5%; BIII) 3%; BIV) 5%; BV) 7%.

Values of strains, loads and morphological parameters from the microCT in situ test are shown in Fig. 4 and listed in Table S6. Load-strain curves (CT*.F* in Fig. 4A and B) were consistent with those of the reference (ex situ) mechanical characterization (Fig. 3C) both for SB and EHS, supporting the validity of the microCT in situ protocol. The measurement of the cross-sectional area of the material during the in situ tensile steps and the net of microporosity, at each strain level, highlighted the expected striction phenomenon (*CT.Cr.Ar* in Fig. 4A and B), allowing also to follow closely the evolution of stress (*CT.σ* in Fig. 4C–I). This revealed that, during microCT acquisition, the SB mechanical response was almost in the yielding region, while EHS was still in the elastic one. The cross-sectional area restriction corresponded to a decrement in microporosity only for SB (intra-bundle voids, *Po* in Fig. 4C) and to an increase for EHS (intra-bundle and inter-bundles voids, *Int.Po* in Fig. 4E), due to the reduction of tortuosity and the parallel increment in separation between bundles with increasing strain. SB tortuosity (*τ* in Fig. 5H) and orientation (*θ* in Fig. 4F) showed no trend with strain and lower average-on-strains values (1.05 and 2°, respectively) with respect to EHS, in which instead they slightly decreased with strain (Fig. 4G and I). This can be related to a stretched structural arrangement in SB, that is in fact yielding, while to a progressive alignment on loading direction in EHS, that is still elastically deforming.

### 3.4 Digital volume correlation analysis

**Fig. 6:**
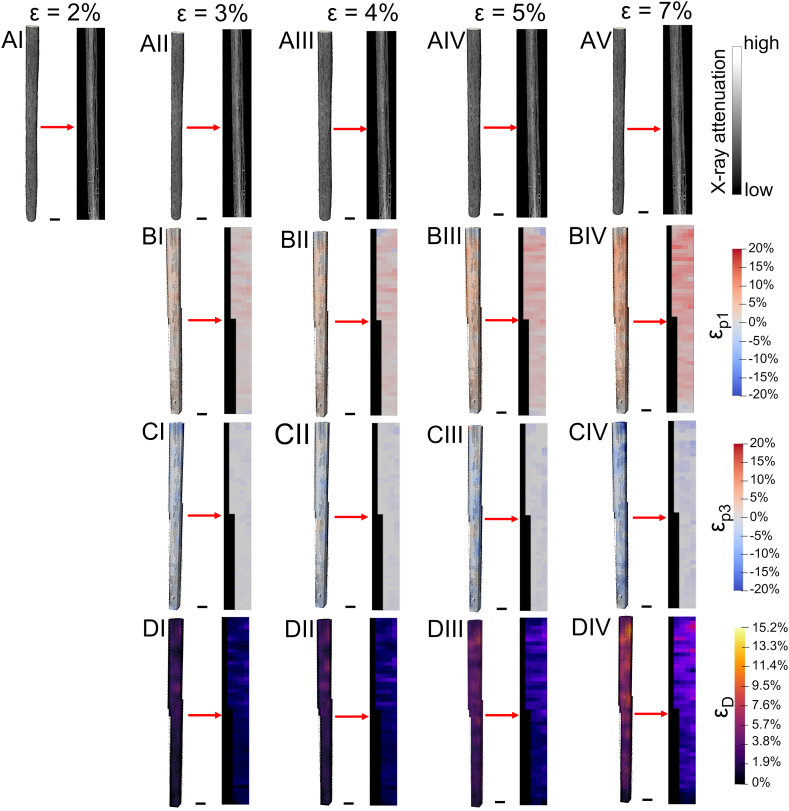
Evolution during the in situ test of the DVC strain fields for a representative SB 3D volume and its internal cross-section (scale bar = 500 μm): A) reconstructed 3D volume renderings and central vertical cross-section for the different strain steps; B) 3D volume renderings and central vertical cross-section for the different strain steps of ε_p1_; C) 3D volume renderings and central vertical cross-section for the different strain steps of ε_p3_; D) 3D volume renderings and central vertical cross-section for the different strain steps of ε_D_.

**Fig. 7:**
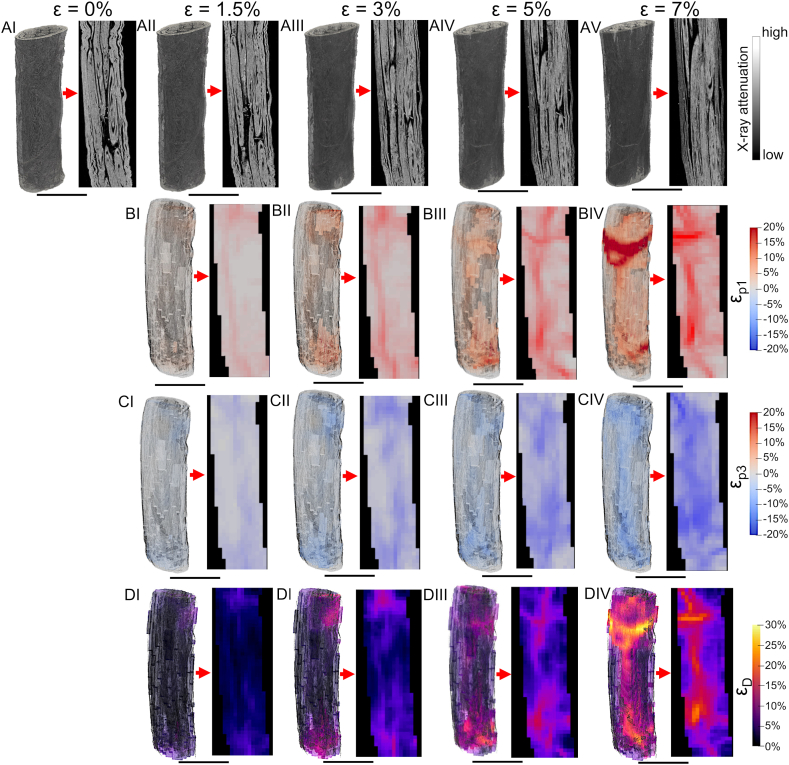
Evolution during the in situ test of the DVC strain fields for a representative EHS 3D volume and its internal cross-section (scale bar = 3 mm): A) reconstructed 3D volume renderings and central vertical cross-section for the different strain steps; B) 3D volume renderings and central vertical cross-section for the different strain steps of ε_p1_; C) 3D volume renderings and central vertical cross-section for the different strain steps of ε_p3_; D) 3D volume renderings and central vertical cross-section for the different strain steps of ε_D_.

The DVC successfully measured, starting from the displacement fields (see Video S1, Video S2), the full-field strain distribution both on SB and EHS (Table 1, Table S7, Table S8, Fig. 6, Fig. 7). The uncertainties calculated where approximately one order of magnitude lower of the mean strain at the yielding point of both SB and EHS. These values are consistent for the strain analysis of such viscoelastic materials.

**Table 1:** Mean ± SD of axial displacements and DVC ε_p1_, ε_p3_, ε_D_ strains between the tested samples of the same category, together with their maximum and minimum values.

Strain Steps (SB)	2–3%	2–4%	2–5%	2–7%
Disp (mm)	0.08 ± 0.03	0.14 ± 0.04	0.21 ± 0.08	0.35 ± 0.12
Disp_max_ (mm)	0.16 ± 0.07	0.25 ± 0.06	0.35 ± 0.12	0.60 ± 0.12
ε_p1_ (%)	1.62 ± 0.30	2.20 ± 0.13	3.05 ± 0.20	4.78 ± 0.56
ε_p1max_ (%)	6.91 ± 2.28	8.43 ± 1.63	11.20 ± 4.12	14.57 ± 6.89
ε_p3_ (%)	−0.97 ± 0.30	−1.01 ± 0.19	−1.36 ± 0.12	−1.85 ± 0.05
ε_p3min_ (%)	−4.28 ± 0.97	−4.50 ± 2.33	−5.24 ± 1.28	−6.11 ± 1.56
ε_D_ (%)	1.92 ± 0.46	2.4 ± 0.26	3.28 ± 0.18	4.86 ± 0.58
ε_Dmax_ (%)	5.13 ± 1.48	6.92 ± 0.94	9.76 ± 2.69	14.12 ± 3.82
**Strain Steps (EHS)**	**0**–**1.5%**	**0**–**3%**	**0**–**5%**	**0**–**7%**
Disp (mm)	0.06 ± 0.03	0.13 ± 0.05	0.23 ± 0.06	0.38 ± 0.06
Disp_max_ (mm)	0.12 ± 0.04	0.22 ± 0.09	0.36 ± 0.09	0.58 ± 0.17
ε_p1_ (%)	2.19 ± 1.23	3.34 ± 1.20	5.15 ± 1.38	7.26 ± 0.75
ε_p1max_ (%)	10.98 ± 3.41	22.74 ± 5.83	30.54 ± 5.67	40.17 ± 3.31
ε_p3_ (%)	−2.41 ± 0.56	−4.10 ± 0.64	−6.27 ± 1.63	−8.35 ± 3.52
ε_p3min_ (%)	−13.06 ± 1.69	−18.50 ± 2.86	−24.90 ± 5.19	−27.00 ± 6.04
ε_D_ (%)	3.35 ± 1.23	5.45 ± 0.34	8.38 ± 0.80	10.75 ± 1.30
ε_Dmax_ (%)	15.22 ± 3.99	22.80 ± 6.33	32.40 ± 3.88	37.77 ± 4.33

A hypothesis of the rearrangement of SB internal nanofibers during the in situ test can be summarized as follows (see Fig. 5, Fig. 6). In the first step the SB are already in the linear region where, having passed the nonlinear toe region, nanofibers are a bit stretched and aligned (Fig. 5AI). At 3% the yielding point is reached, and nanofibers, and the wrapped mat layers that compose each SB, rise their stretching and ε_p1_ (Fig. 5AII). The first ruptures of nanofibers and layers occur, enhancing ε_D_, while nanofibers progressively reduce their diameters, showing some relaxation. These phenomena increase the negative regions and ε_p3_ also causing an amplification of sliding (ε_D_) in the subsequent strain steps, progressively amplifying the phenomena previously described (Fig. 5AIII-5AV).

Specifically, SB (Table 1, Fig. 6 and Table S7) (see Fig. 6AI-AV for typical SB microCT renderings at the different strain steps) showed, as expected, increasing ε_p1_ strains during the in situ test reaching local values of ε_p1_ = 4.78 ± 0.56% at 7% step, but with maximum peak of ε_p1max_ = 14.57 ± 6.98% (from 10 up to 3 times higher strain compared to the apparent strain values) (Fig. 6B). Consistently, ε_p3_ confirmed a progressive striction of the cross-section of SB with the elongation/yielding of the internal nanofibers with mean negative values of ε_p3_ = −1.85 ± 0.05% (ε_p3min_ = −6.11 ± 1.56%) at 7% step (Fig. 6C). However locally, SB also exhibited positive values of ε_p3_ that could be probably caused by the concomitant presence of: i) internal reorganization of the nanofibers and layers of the original electrospun mat used for generating the bundles; ii) local relaxation of groups of nanofibers as they yield. Similarly, the deviatoric strain ε_D_ confirmed a progressive sliding of the internal electrospun layers of the wrapped mat of bundles, their nanofibers and the evolution of the internal porosities of SB, reaching mean values of ε_D_ = 4.86 ± 0.58% (Fig. 6D), with local maxima ε_Dmax_ = 14.12 ± 3.82%. Considering the PLLA/Coll nanofibers, these were not resolvable at the voxel size achieved from microCT (i.e. SB = 13 μm; EHS = 9 μm). However, their overall rearrangement inside the SB volume can explain the strain behavior of SB, which is also supported by the morphometric investigation (see Fig. 4 and Table S6).

For EHS instead, the strain evolution must consider additional phenomena (Figs. 5B and 7) (see Fig. 7AI-AV for typical EHS microCT renderings at the different strain steps). At the reference step in this case, the nanofibers and bundles are waved, being in the toe region of scaffolds (this happens because the initial pre-load is distributed in the internal bundles), while the nanofibers in the membrane are partially circumferentially oriented (Fig.5BI). In the first step, nanofibers and bundles progressively increase their alignment producing some sliding and tightening of the membrane and causing an increment of ε_D_ and ε_p1_ (Fig. 5BII). In the second step, nanofibers and bundles are now aligned experiencing an incremental stretching/sliding of the internal bundles and nanofibers, and with a parallel tightening of the membrane (with a rise of ε_D_ and ε_p1_) (Fig. 5BIII). These phenomena will also cause an overall reduction of EHS diameter (increment of ε_D_ and decrement of ε_p3_) but also some yielding of the smallest nanofibers (ε_p1_ and ε_p3_ up). In the last steps, being EHS close to yielding, all the previous phenomena are progressively amplified including the rupture of bunches of nanofibers (raising all the strains) (Fig. 5BIV and 5BV).

More specifically, in EHS (Fig. 7 and Table 1 and Table S8), the strain-guided evolution of the internal bundles and void spaces were detected from the images. The ε_p1_ showed a progressive increment of mean values up to ε_p1_ = 7.26 ± 0.75% (ε_p1max_ = 40.17 ± 3.31%) at 7% of apparent strain. EHS also showed negative regions with a mean ε_p3_ = −8.35 ± 3.52% at 7% step (ε_p3min_ = −27.00 ± 6.04%) (Fig. 7B), suggesting a progressive compaction and reorganization of the internal bundles and nanofibers of the membrane. Moreover, due to their reorganization during the different strain steps, the preferential transversal alignment of the nanofibers of the membrane contributed to a progressive tightening of the EHS, increasing the negative values of ε_p3_ (Fig. 7C). These data are also in accordance with the morphometric increment of internal porosity and θ of EHS, the progressive decrement of (τ) (Fig. 4 and Table S6) and the slightly increment of the transversal strain of the membranes between ε_Y_ and the 7% step (Fig. 3G). All these adjustments of EHS internal bundles were also confirmed by the mean values of ε_D_ = 10.75 ± 1.30% (Fig. 7D) in correspondence of their maximum sliding (ε_Dmax_ = 10.75 ± 1.30%).

The DVC full-field strain distribution of SB and EHS produced a strain behavior similar to that experienced in the natural T/L tissue counterpart by using DVC [43], DIC [[25], [26], [27], [28], [29], [30],^61^,^62^] and finite element models [63]. The progressive stretching and reorganization of internal collagen fibrils/fascicles of T/L during physiological activities is responsible for the nonlinear behavior of their stress/strain curves. This characteristic is typically visible using DIC on T/L and resulting into inhomogeneous strain patterns that follow the local stretch/relaxation during tensile test, reaching mean values from 8% up to 25% depending on the T/L under investigation [[25], [26], [27], [28], [29], [30],^61^,^62^]. The inhomogeneous strain patterns were due to the internal rearrangement, and progressive failure, of groups of collagen fibrils of the T/L of interest. Moreover, it is also well established that the collagen fibrils in T/L start damaging in the linear region of the stress/strain curve in a specific point defined as inflection point [51]. In this study, the inflection strain of SB was IPε = 2.12 ± 0.57, while for EHS was IPε = 6.66 ± 3.12. These explain the large increment of ε_p1max_ at the strain steps corresponding to the yielding point of SB (strain step 3%) and EHS (strain step 7%) where scaffolds/nanofibers locally start yielding earlier than the macroscopic strain point, similarly to natural T/L [51]. This confirms the importance of DVC measurements in the characterization of hierarchical materials for T/L regeneration. Overall, this behavior of internal yield/relaxation in both SB and EHS from is therefore consistent with natural T/L and their fascicles.

The measured full-field mechanics of the examined scaffolds contributed to better explain the morphological changes and elongation of fibroblasts and tenocytes previously detected during static [13,14] and dynamic cultures in bioreactor [18]. These data contribute to explain the reason why these scaffolds can interact this cells guiding their morphological changes in shape and orientation [18].

This study confirmed the promising morphological and mechanical performance of such scaffolds for T/L tissue engineering, also showing some limitations mostly related to the resolution of the microCT scans and the clamping setup. In fact, as mentioned, the resolution of the microCT system used in this study did not allow detection of individual nanofibers, as well as their internal micro/nano porosities much smaller than the nominal voxel size (scans voxel size = 9–13 μm). Also, the step-wise nature of the in situ test (i.e. 30 min for each strain step) contributed to partial relaxation inside the scaffolds due to the intrinsic viscoelasticity of these polymeric materials. All samples of SB and EHS showed peaks of strain in close proximity of the clamps of the in situ loading device confirming, as expected, strain concentrations due to the clamping setup. Future studies will require reduced scanning times coupled with higher resolutions, for example using synchrotron x-ray computed tomography, to visualize and measure phenomena at the nanofiber and using dedicated capstan grips to minimize strain concentration.

## 4 Conclusion

In this study, biomimetic PLLA/Coll-based electrospun scaffolds for T/L tissue engineering were successfully produced and characterized with techniques such as in situ microCT and DVC allowing, for the first time, to measure the 3D volumetric full-field strain distribution of such electrospun materials. The scaffolds mimicked the multiscale morphological and mechanical behavior of the natural collagen fibril/fascicles to the whole T/L tissue. The combination of in situ microCT mechanics and DVC achieved in this study will provide fundamental insights for future research on electrospinning and regenerative medicine, to better understand the complex interplay between nanofibrous structure/mechanics and how this can optimally drive cell fate in vivo.

## Data availability

Data will be made available on request.

## CRediT authorship contribution statement

**Alberto Sensini:** Writing – review & editing, Writing – original draft, Visualization, Project administration, Methodology, Investigation, Funding acquisition, Formal analysis, Data curation, Conceptualization. **Olga Stamati:** Writing – review & editing, Writing – original draft, Visualization, Methodology, Investigation, Formal analysis. **Gregorio Marchiori:** Writing – review & editing, Writing – original draft, Methodology, Investigation, Formal analysis. **Nicola Sancisi:** Writing – review & editing, Writing – original draft, Methodology, Investigation, Formal analysis. **Carlo Gotti:** Writing – review & editing, Writing – original draft, Investigation, Formal analysis. **Gianluca Giavaresi:** Writing – review & editing, Supervision. **Luca Cristofolini:** Writing – review & editing, Writing – original draft. **Maria Letizia Focarete:** Writing – review & editing, Writing – original draft. **Andrea Zucchelli:** Writing – review & editing, Writing – original draft, Supervision, Methodology, Funding acquisition, Conceptualization. **Gianluca Tozzi:** Writing – review & editing, Writing – original draft, Supervision, Methodology, Conceptualization.

## Declaration of competing interest

The authors declare the following financial interests/personal relationships which may be considered as potential competing interests: Alberto Sensini has patent HIERARCHICAL MULTISCALE ELECTROSPUN SCAFFOLD FOR THE REGENERATION AND/OR REPLACEMENT OF THE TENDINOUS/LIGAMENTOUS TISSUE AND A METHOD FOR ITS PRODUCTION issued to Alma Mater Studiorum - Università di Bologna. Andrea Zucchelli has patent HIERARCHICAL MULTISCALE ELECTROSPUN SCAFFOLD FOR THE REGENERATION AND/OR REPLACEMENT OF THE TENDINOUS/LIGAMENTOUS TISSUE AND A METHOD FOR ITS PRODUCTION issued to Alma Mater Studiorum - Università di Bologna. Luca Cristofolini has patent HIERARCHICAL MULTISCALE ELECTROSPUN SCAFFOLD FOR THE REGENERATION AND/OR REPLACEMENT OF THE TENDINOUS/LIGAMENTOUS TISSUE AND A METHOD FOR ITS PRODUCTION issued to Alma Mater Studiorum - Università di Bologna. Maria Letizia Focarete has patent HIERARCHICAL MULTISCALE ELECTROSPUN SCAFFOLD FOR THE REGENERATION AND/OR REPLACEMENT OF THE TENDINOUS/LIGAMENTOUS TISSUE AND A METHOD FOR ITS PRODUCTION issued to Alma Mater Studiorum - Università di Bologna. If there are other authors, they declare that they have no known competing financial interests or personal relationships that could have appeared to influence the work reported in this paper.
